# Apathetic Thyroid Storm with Cardiorespiratory Failure, Pulmonary Embolism, and Coagulopathy in a Young Male with Graves' Disease and Myopathy

**DOI:** 10.1155/2020/8896777

**Published:** 2020-09-23

**Authors:** Abuobeida Ali, Wail Mostafa, Cornelius Fernandez, Habib Ahmad, Nyi Htwe

**Affiliations:** ^1^Department of Acute Medicine, Pilgrim Hospital, Boston PE21 9QS, UK; ^2^Department of Gastroenterology, Pilgrim Hospital, Boston PE21 9QS, UK; ^3^Department of Endocrinology, Pilgrim Hospital, Boston PE21 9QS, UK

## Abstract

A 38-year-old gentleman presented with thyroid storm with multiorgan involvement in the form of heart failure (thyrotoxic cardiomyopathy), respiratory failure (respiratory muscle fatigue), hepatic dysfunction, fast atrial fibrillation, pulmonary embolism, and disseminated intravascular coagulation (DIC). His Graves' disease (GD) remained undiagnosed for nearly 8 months because apart from weight loss, he has not had any other symptoms of thyrotoxicosis. The presentation of thyroid storm was atypical (apathetic thyroid storm) with features of depression and extreme lethargy without any fever, anxiety, agitation, or seizure. There were no identifiable triggers for the thyroid storm. Apart from mechanical ventilation and continuous veno-venous renal replacement therapy in the intensive care unit, he received propylthiouracil (PTU), esmolol, and corticosteroids, which were later switched to carbimazole and propranolol with steroids being tapered down. He was diagnosed with thyrotoxic myopathy which, like GD, remained undiagnosed for long (fatigability). A high index of suspicion and a multidisciplinary care are essential for good outcome in these patients.

## 1. Case Presentation

A 38-year-old gentleman presented to the emergency department (ED) with worsening shortness of breath, chest tightness, and dry cough of 3-day duration. Apart from these symptoms, he has been experiencing frequent watery diarrhoea for 2 weeks associated with intermittent vomiting, fatigue, extreme lethargy, and a depressed mood. He has had a weight loss of nearly 6-7 kg over 8 months, which the patient considered as intentional. There was no history of fever, palpitation, abdominal pain, haematemesis, malaena, jaundice, loss of appetite, agitation, confusion, or seizure. He had a past medical history of bronchial asthma, well controlled on a salbutamol inhaler. Social history revealed a high-risk sexual behaviour that has resulted in a penile sore which was investigated in the genitourinary outpatient clinic. While in ED, he has had an episode of frank haemoptysis.

On examination, he was alert, but ill looking, cachectic, dyspnoeic, and tachypneic. Observations were as follows: pulse rate of 151 beats/minute, blood pressure (BP) of 109/82 mmHg, respiratory rate of 28/minute, oxygen saturation (SPO_2_) of 98% on 2 litres of oxygen, and temperature of 36.2 C. Cardiorespiratory examination showed grossly congested neck veins, with bibasal crackles. His Glasgow Coma Scale (GCS) was 15/15, and there was no focal neurological deficit. His abdomen was soft, mildly distended, with normal bowel sounds. He had no lower limb oedema or deep vein thrombosis. Genital examination showed penile cellulitis which has been an ongoing problem for last few weeks.

## 2. Investigations

**Figure 1:**
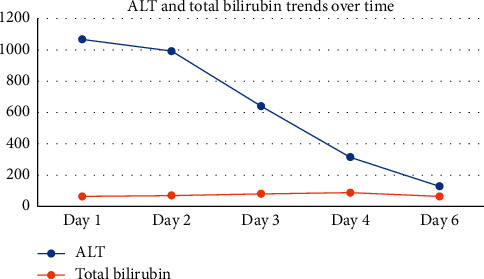
Trends of ALT and total bilirubin in the first 6 days of hospitalisation.

**Figure 2:**
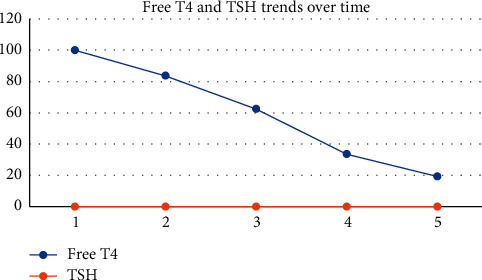
Trends of free T4 and TSH in the first 6 days of hospitalisation.

**Figure 3:**
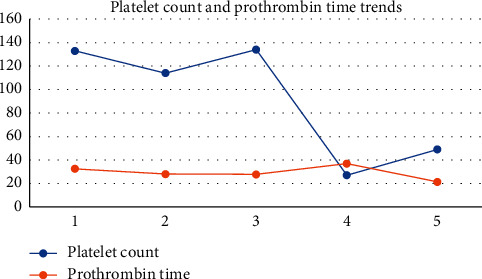
Trends of platelet count and prothrombin time in the first 6 days of hospitalisation.

ECG showed evidence of atrial fibrillation (AF) with a ventricular rate of 155 beats/minute. The arterial blood gas done on 2 litres of oxygen showed a pH of 7.392 (7.35–7.45), PO_2_ of 10.43 kPa (10.67–13.33), PCO_2_ of 2.88 kPa (4.67–6), lactate of 3.9 mmol/L (1–2.5), bicarbonate of 12.9 mmol/L (22–28), and base excess −10.01 mmol/L (−2 to +2). Bedsides, echo done by FAST (focused assessment with sonography for trauma) by a trained emergency physician revealed right ventricular dilation. Hence, a provisional diagnosis of possible pulmonary embolism (PE), with PE triggered AF, was considered. However, in view of cachexia, underlying malignancy or immunodeficiency was to be excluded. The serial blood results are given in Figures 1–3.

CT pulmonary angiogram along with CT abdomen-pelvis was done which revealed evidence of acute peripheral pulmonary embolism involving the segmental and subsegmental branches of the right lower lobe pulmonary artery associated with significant cardiomegaly, bilateral moderate basal pleural effusion, congestive hepatomegaly, mild-to-moderate ascites, and significant subcutaneous oedema in the abdominopelvic region. In view of the AF and CT evidence of heart failure, additional tests like the thyroid function test and NT‐proBNP (*N*-terminal probrain natriuretic peptide) were sent. He received intravenous digoxin and furosemide with therapeutic dose of enoxaparin.

## 3. Diagnosis and Treatment

His general condition rapidly deteriorated with respiratory muscle fatigue and an impending respiratory arrest. Hence, he was transferred to the intensive care unit (ICU). He rapidly became haemodynamically unstable with profound metabolic acidosis necessitating electrical cardioversion (for fast AF with haemodynamic compromise), rapid sequence intubation, and mechanical ventilation. The cardioversion successfully reverted AF to sinus tachycardia. His profound metabolic acidosis and fluid overload (heart failure) were treated with continuous veno-venous renal replacement therapy (CRRT).

His additional blood tests showed TSH <0.01 mU/L (0.27–4.5), free T4 >100 pmol/L (11–23), free T3 10.16 pmol/L (3.1–6.8), and NT‐proBNP 19,398 ng/L (0–300). Accordingly, the ITU team has started the patient on carbimazole and hydrocortisone with a diagnosis of thyrotoxic crisis with AF. Patient's liver function got worse even before starting carbimazole. Hence, it was attributed to congestive hepatomegaly. A diagnosis of thyroid storm with the Burch–Wartofsky Point Scale (BWPS) of 80, heart failure secondary to cardiomyopathy, and deranged liver function tests secondary to congestive hepatomegaly were agreed by the endocrine team. They suggested to switch carbimazole to propylthiouracil (PTU) and hydrocortisone to dexamethasone. Additionally, they advised to seek the cardiology opinion to choose the most appropriate *β*-blocker in view of heart failure and to do the thyroid peroxidase antibody (TPO), TSH receptor antibody (TRAb), thyroid ultrasound, and formal echocardiogram (ECHO).

The formal ECHO showed that the left ventricle was moderately dilated with severe global hypokinesia with an ejection fraction (EF) of 27%. Right ventricle was of normal size, but with severely impaired systolic function. The gastroenterologist and cardiologist agreed with the diagnosis of congestive heart failure secondary to thyrotoxic cardiomyopathy and deranged liver function tests caused by congestive hepatomegaly. Tests for HIV, *Treponema pallidum*, covid-19, and tuberculosis were negative. The cardiologist suggested to use esmolol until stable thyroid and cardiac functions are achieved and then to switch to propranolol 20 mg three times daily. A follow-up ECHO repeated 2 weeks later showed an improvement in EF to 36%. Infective hepatitis screen and autoimmune liver screen were negative.

Thyroid ultrasound demonstrated a bulky thyroid with retrosternal extension and heterogeneous echotexture, with moderately increased vascularity suggestive of thyroiditis. A review of the CT pulmonary angiogram by the radiologist revealed a disproportionately enlarged left thyroid lobe, with mild tracheal deviation towards the right. The TRAb came as 8.16 U/L (0–1.74) and TPO came as 593 IU/ml (0–34). On the 5^th^ day of admission, his free T4 was 19.3 pmol/L (11–23), free T3 was 2.94 pmol/L (3.1–6.8), and TSH was <0.01 mU/L (0.27–4.5). The endocrinology team advised to continue PTU for a total of 6 weeks and then switch to carbimazole.

During the ICU stay, he had persistent thrombocytopenia (lowest 27 × 109/L), persistently prolonged prothrombin time (highest 36.9 seconds), occasionally prolonged activated partial thromboplastin time (highest 40.2 seconds), with persistently high D-dimer levels (highest 6,713 ng/mL), and a reduced Clauss fibrinogen level of 1.4 g/L. On the International Society on Thrombosis and Haemostasis (ISTH) criteria for DIC, he scored 7 which was compatible with a diagnosis of DIC, and he was treated with frozen plasma and vitamin K under haematology supervision. He developed a hospital-acquired pneumonia with *Pseudomonas* grown on the sputum culture and received piperacillin-tazobactam and nebulised colomycin with microbiology input.

## 4. Outcome of Treatment

In two weeks, he was extubated and stepped down to the medical ward, where he was found to have evidence of significant proximal myopathy associated with a significant difficulty in swallowing. Assessment by the Speech and Language Therapy (SALT) team observed an evidence of severe oesophageal dysphagia. He was on nasogastric feeding due to high risk of aspiration pneumonia. For the neuromuscular issues, he was seen by the neurologist who has made a provisional diagnosis of thyrotoxic myopathy which was later confirmed by electromyography. He is currently receiving physiotherapy support for his muscle weakness, and at the time of writing this case report, his muscle power was slowly improving. His swallowing has already improved, and he is able to tolerate oral feeding. The endocrine team has planned to continue carbimazole (CBZ) for a period of 12–18 months, and if the hyperthyroidism relapses, he will be considered for radioactive iodine (RAI) or thyroidectomy.

## 5. Discussion

### 5.1. Introduction

Thyroid storm (TS), also known as thyrotoxic crisis or thyroid crisis, is a life-threatening hypermetabolic thyrotoxicosis, presenting as multiorgan dysfunction with or without a known precipitating cause [1]. This is a rare condition with an annual incidence of 0.57–0.76/100,000 persons in community residents, whereas among hospitalized patients, the annual incidence is 4.8–5.6/100,000 patients [1]. Thyroid storm is associated with 12-fold higher hospital mortality compared to thyrotoxicosis without storm (1.2–3.6% vs. 0.1–0.4%), longer hospital stays (4.8–5.6 vs. 2.7–3.4 mean days), and increased treatment costs [1].

Various physical or mental stressors can act as triggers to induce thyroid storm in patients with diagnosed or undiagnosed thyrotoxicosis [2]. Common triggers include infection, acute illness (myocardial infarction, stroke, hypoglycaemia, and diabetic ketoacidosis), emotional stress, noncompliance with antithyroid drugs, thyroidectomy, neck trauma (strangulation), nonthyroidal surgery, pregnancy, delivery, iodinated contrast, high-dose iodine, and radio-iodine treatment [2]. Rare triggers include thyroid palpation, thyroid fine needle aspiration, subacute thyroiditis, thyroid hormone over dosage, metastatic thyroid cancer, gestational trophoblastic disease, struma ovarii, intense exercise, and finally drugs including anaesthetics, salicylates, pseudoephedrine, amiodarone, *α*-interferon, interleukin-2, methamphetamine, sorafenib, and ipilimumab. In nearly 25–45% of patients, no definite trigger is identified [2].

### 5.2. Pathophysiology

Thyroid storm occurs in 1-2% of subjects with untreated/poorly controlled hyperthyroidism. Thyroid storm is commonly associated with Graves' disease. Nevertheless, it can happen with toxic adenoma or toxic multinodular goitre [2]. Even though uncertain, the pathophysiology of thyroid storm is thought to be due to an acutely increased release, decreased metabolism (increasing intracellular levels), decreased plasma protein binding (increasing free hormone levels), or augmented peripheral response of thyroid hormones, activation of the *β*-adrenergic system, and relative adrenal insufficiency caused by the hypermetabolic state [3]. The thyroid function tests in thyrotoxic subjects with storm are not different from those without storm, and there are no absolute thyroid hormone level cutoffs to diagnose thyroid storm [3].

### 5.3. Clinical Features and Diagnosis

The diagnosis of thyroid storm is purely clinical, and an accurate diagnosis is crucial to improve the morbidity and mortality. The clinical features of thyroid storm are that of an exaggerated hyperthyroidism associated with multiorgan dysfunction [2]. The Burch–Wartofsky Point Scale (BWPS) proposed in 1993 for diagnosis of thyroid storm provides a quantitative diagnostic aid where increasing points are given for greater severity of dysfunction [4]. According to the BWPS (Table 1), a score ≥45 provides a diagnosis of thyroid storm, a score of 25–44 suggests impending thyroid storm, and a score <25 makes the thyroid storm unlikely [4].

Another nonquantitative criteria proposed by the Japan Thyroid Association known as Akamizu criteria (Table 2) categorize patients into definite or suspected thyroid storm based on presence of defined clinical features [6]. It is advised to use both criteria for the accurate diagnosis [5]. Nevertheless, inappropriate use of either can lead to misdiagnosis [5]. The BWPS has superior sensitivity for diagnosing thyroid storm compared to the Akamizu criteria [7], but it is less specific for the diagnosis [8]. The thyroid storm patients with CNS dysfunction derive the greatest benefit from aggressive treatment [8]. The diagnosis of apathetic thyroid storm (thyroid storm without fever and agitation) is challenging. Though apathetic thyroid storm is a disease of elderly [9], there are occasional case reports in young adults [10] and children [11].

According to the Akamizu criteria, thyroid storm should not be considered if there are other underlying diseases that can clearly explain the symptoms. However, if a physician cannot accurately differentiate the exact origin of symptoms, whether from trigger or thyroid storm, consider that symptoms are due to thyroid storm caused by the trigger and the treatment should be given for both. For example, in a patient with antithyroid drug- (ATD-) associated agranulocytosis, the symptoms could either be due to sepsis [12] or be due to the thyroid storm triggered by sepsis [13]. Here, treatment should be given for both sepsis and thyroid storm [12].

### 5.4. Cardiac Failure and Cardiomyopathy

Thyrotoxicosis could have both central and peripheral effects on the cardiovascular system [14]. The central effects include direct effect on the sinoatrial node causing atrial tachyarrhythmias and direct effect on the heart muscle causing increased myocardial contractility. In the peripheral circulation, it causes vasodilatation leading to a fall in peripheral vascular resistance, after load and diastolic blood pressure. Reduction in renal perfusion secondary to vasodilatation causes an activation of the renin-angiotensin-aldosterone system (RAAS) with resultant sodium retention, increase in circulating volume, preload, and stroke volume. The increase in myocardial contractility associated with decrease in peripheral vascular resistance leads to an increase in cardiac output (2-3-fold) and systolic hypertension with a wide pulse pressure [14].

When the circulating volume significantly increases, it decreases the myocardial contractile reserve leading on to high-output heart failure [15] (HF). Nearly, 6% of thyrotoxic patients would develop high-output HF and 1% would develop thyrotoxic cardiomyopathy (TCMP), a form of dilated cardiomyopathy having low cardiac output with impaired left ventricular systolic and diastolic functions [14]. Mechanisms for TCMP apart from the direct effect of tachycardia include autoimmune or lymphocytic myocarditis [16]. Rarely, stress-induced reversible cardiomyopathy (Takotsubo) characterized by apical hypokinesia has been reported with thyrotoxicosis [17]. Isolated right ventricular dysfunction with pulmonary hypertension has been reported with thyrotoxicosis. An explanation is the increased cardiac output increasing the right ventricular preload, which in turn causing pulmonary vascular endothelial sheer stress, stimulating pulmonary vasoconstriction, pulmonary hypertension, and right ventricular dysfunction [18].

#### 5.4.1. Respiratory Fatigue/Failure

Inspiratory muscle fatigue and failure causing respiratory acidosis, necessitating mechanical ventilation, is not uncommon with thyroid storm, as thyrotoxicosis is known to decrease the muscle mass by 20% and muscle strength by 40% [19]. Various mechanisms for acute respiratory failure include pulmonary oedema [19], pulmonary embolism [20], thyrotoxic myopathy, thyrotoxic periodic paralysis, rhabdomyolysis, polymyositis, or coexisting myasthenia gravis [21].

#### 5.4.2. Renal Failure and Rhabdomyolysis

Renal failure could be due to hypoperfusion, infection, or rhabdomyolysis, with the latter due to fever, agitation, hypermetabolism, hypoperfusion, hypokalaemia, infection, or steroids [21

#### 5.4.3. Venous and Arterial Thromboembolism

Thyrotoxicosis is a hypercoagulable state characterized by increased fibrinogen, FVIII, and vWF activity (due to a direct effect on gene transcription of coagulation proteins) associated with decreased fibrinolysis [22]. Other mechanisms include contact system activation, neutrophil extracellular trap formation, or immune-mediated inflammatory response [22]. Thyroid storm could be associated with deep vein thrombosis with pulmonary embolism [23], cerebral venous sinus thrombosis [24], or extensive systemic venous thromboembolism [25]. It could also cause ischaemic stroke by arterial thrombosis or by embolism from atrial fibrillation [26].

#### 5.4.4. Atrial Fibrillation

AF has a prevalence of 10–20% in thyrotoxicosis and 30–40% in thyroid storm [23]. Even though thyroid storm is a hypercoagulable state, there is no consensus regarding therapeutic anticoagulation even when thyroid storm is associated with AF, as thyrotoxicosis is not included in the CHA_2_DS_2_-VASc score. However, few authors have recommended that anticoagulation should be considered in patients with thyroid storm or impending storm irrespective of the CHA_2_DS_2_-VASc score [23]. Moreover, few others have recommended that anticoagulation should be considered in all cases of thyroid storm even in the absence of AF [25].

#### 5.4.5. Hepatic Manifestations

Deranged liver function tests in thyroid storm could be due to multiple mechanisms including heart failure-induced congestive hepatomegaly [27], peripheral vasodilatation-induced hepatic ischaemia [27], thyrotoxicosis-induced direct hepatocyte toxicity [27], concomitant autoimmune hepatic diseases (autoimmune hepatitis and primary biliary cirrhosis) [27], or effect of antithyroid drugs [28]. Hyperthyroidism as the cause of deranged liver function can only be considered after all those aetiologies are ruled out [29

Congestive hepatomegaly is associated with moderate (2- or 3-fold) transaminase levels with bilirubin levels that are rarely >50 *μ*mol/L, whereas ischaemic hepatitis is associated with extremely high bilirubin (∼250 *μ*mol/L) and transaminase (>10-fold) levels [30]. Fulminant hepatitis is a rare, life-threatening complication of thyroid storm associated with multiorgan failure and poor prognosis [27]. Orthotopic liver transplantation is the treatment of choice for thyroid storm-associated fulminant hepatitis [27].

#### 5.4.6. Haematological Manifestations

In the euthyroid state, thyroid hormones stimulate haematopoietic stem cells increasing blood cell formation. Thyrotoxicosis causes anaemia (34%), leukopenia (5.8%), and thrombocytopenia (3.3%). But, pancytopenia is rare [30]. Mechanisms include thyrotoxicosis-induced direct bone marrow toxicity, ineffective haematopoiesis [31], or reduced lifespan of blood components by stimulation of the reticuloendothelial system [32], autoimmune mechanisms [32] (pernicious anaemia and immune thrombocytopenic purpura), or by a *β*-adrenergic mechanism [32]. Moreover, thionamides are associated with leucopenia or agranulocytosis. Direct bone marrow toxicity of the thyroid storm is suggested when thionamide improves leucopenia. Though many triggers for thyroid storm including infection, surgery, and trauma can provoke DIC (disseminated intravascular coagulation), thyroid storm could directly cause it through systemic inflammatory response syndrome. The resultant DIC could worsen the thrombocytopenia caused by thyroid storm [33].

#### 5.4.7. Neurological Manifestations

Though commonly associated with acute confusion [34] or agitation [34] which resolves within 2 weeks after normalization of thyroid function [35], thyroid storm might present with protracted loss of consciousness [35], psychosis [36], status epilepticus [37], extreme lethargy [10], or coma [38].

### 5.5. Treatment of Thyroid Storm

A multidisciplinary care, with specialist inputs from endocrinology, cardiology, neurology, hepatology, and haematology, is needed in the management of thyroid storm [3]. The treatment targets involve biochemical control of thyrotoxicosis, control of symptoms and signs, control of multiorgan involvement, treatment of triggers, and definitive treatment of thyrotoxicosis [3]. The various treatment modalities involve drugs like antithyroid drugs, corticosteroids, beta-blockers, and inorganic iodine (Table 3), along with volume resuscitation, aggressive cooling with antipyretics or cooling blankets, nutritional support, respiratory care, and ICU monitoring [39].

#### 5.5.1. Antithyroid Drugs (ATDs)

The ATDs are carbimazole (CBZ), methimazole (MMI), and propylthiouracil (PTU). MMI is the active metabolite of CBZ [40]. CBZ/MMI is at least 10 times potent than PTU [40]. Moreover, MMI has longer half-life (6–8 hours vs. 90 minutes) and duration of action (>24 hours vs. 8–12 hours), compared to PTU [41]. This enables once daily administration of CBZ/MMI, whereas PTU needs multiple daily dosing [41]. PTU is the preferred ATD in thyroid storm due to additional inhibition of peripheral deiodinase-mediated T4 to T3 conversion [42]. When used in standard doses, ATDs could cause agranulocytosis (0.2–0.5%), hepatic dysfunction (0.03% for CBZ/MMI and 0.05% for PTU), rash (6% for CBZ/MMI and 3% for PTU), antineutrophilic cytoplasmic antibody positive vasculitis (PTU), and antithyroid arthritis syndrome (CBZ/MMI) [40].

Both agranulocytosis and hepatic dysfunction are dose related for CBZ/MMI but not for PTU [40]. The high doses that are required in thyroid storm are unlikely to cause harm with PTU than with CBZ/MMI. As there is 15.2% cross-reaction between CBZ and PTU for agranulocytosis, patients with PTU-induced agranulocytosis should not be given CBZ/MMI, and vice versa. In such scenarios, radioactive iodine (RAI) or thyroidectomy should be considered. Transaminase elevations are seen in one-third of thyrotoxicosis patients [40]. A hepatitic pattern is seen with PTU, whereas a cholestatic pattern occurs with CBZ/MMI [40]. Worsening of transaminase levels or new transaminase elevation that is >3 times the upper limit of normal is an indication to stop the ATD. Though severe cases of PTU-induced hepatic dysfunction should be considered for RAI or thyroidectomy, mild cases can be managed by a switch to CBZ/MMI [43]. Though PTU is known to worsen the hepatic dysfunction of thyroid storm, it is still the preferred drug due to added effect [42]. But, careful liver function monitoring is indicated.

#### 5.5.2. Beta-Blockers

Thyroid hormones increase the *β*-adrenergic receptor density by increased formation and decreased degradation [44]. Hyperthyroidism has increased sensitivity to catecholamine and increased sympathetic tone. Noncardio selective beta-blockers (NCBB) like propranolol were traditionally used in thyroid crisis as they could not only overcome the hyperadrenergic state and control the peripheral symptoms, but also could block the T4 to T3 conversion [44]. Thyroid storm patients with clinical or subclinical TCMP might develop an exaggerated response to propranolol resulting in cardiogenic shock [44]. Hence, it is advisable to use intravenous cardio selective *β*-blockers like esmolol (short-acting) or landiolol (ultrashort-acting) that would allow easy titration and rapid cessation of *β*-blocking effect without the risk of prolonged cardiac depression [44]. The half-life and *β*1/*β*2 selectivity for esmolol versus landiolol were 9.19 minutes versus 3.96 minutes and 33 versus 255, respectively [45]. Bisoprolol is preferred over propranolol for tachycardia in thyroid storm [5]. It is preferable to use either PTU or inorganic iodides for inhibiting T4 ⟶ T3 conversion rather than using NCBB for this purpose.

#### 5.5.3. Inorganic Iodide

Acting through the Wolff–Chaikoff effect, inorganic iodide (saturated solution of potassium iodide or SSKI as well as Lugol's solution) reduces the thyroid hormone much faster than ATDs and corticosteroids [5]. However, their effect might disappear after 1-2 weeks in some patients [5]. Inorganic iodide can either be given 1 hour after [40] or simultaneously with [5] ATD administration. Once stable, the inorganic iodide should be reduced before tapering dose of ATD [5].

#### 5.5.4. Cholestyramine

Thyrotoxicosis is associated with an abnormal increase in enterohepatic circulation of thyroid hormones [46]. Cholestyramine could bind these thyroid hormones and remove them from the enterohepatic circulation [46]. The drug could be used in refractory hyperthyroidism, iodine-induced hyperthyroidism, and in whom the ATDs are contraindicated [46].

#### 5.5.5. Corticosteroids

Corticosteroids, in large doses, inhibit thyroid hormone release, block conversion to T4 to T3, promote vascular stability, and prevent relative adrenal insufficiency which is associated with the hypermetabolic state of thyroid storm. Hydrocortisone and dexamethasone, the commonly used corticosteroids in thyroid storm, are associated with improved survival [47]. Corticosteroids should be continued until resolution of thyroid storm. The corticosteroid dose should be appropriately tapered depending on the duration of the corticosteroid therapy. In those who were on corticosteroids for long duration, the drug should be stopped only after confirming that adrenals have recovered [47]. Dexamethasone is preferred by some authors due to its less frequent dosing and availability of intravenous, oral, and nasogastric formulations [48]. Hydrocortisone is preferred by few as it has both mineralocorticoid and glucocorticoid effects (1 : 1), whereas dexamethasone has only a negligible mineralocorticoid effect [49]. Inconsistency in recommendations exists even in standard pharmacology text books [50]. Hence, as per latest papers, either dexamethasone or hydrocortisone can be used in patients with thyroid storm [5].

#### 5.5.6. Therapeutic Plasma Exchange (TPE)

TPE improves the thyroid storm by rapidly removing the large molecular weight substances like thyroid hormones, TSH receptor antibodies, catecholamines, and cytokines [51]. Similarly, TPE would help to replace the thyroid-binding proteins. Fresh frozen plasma (FFP) is preferred as the replacement fluids compared to albumin, as it contains higher levels of TBG to bind to thyroid hormones [51]. Absolute indication for TPE is acute liver failure associated with thyroid storm, whereas relative indication is lack of clinical improvement after 24–48 hours of ATDs, *β*-blockers, corticosteroids, and inorganic iodide, treatment of triggers, and complications [51]. Moreover, when ATDs are not effective or contraindicated, TPE is used as a bridge to control the thyroid hormones until the curative treatment is introduced [5].

#### 5.5.7. Radioactive Iodine (RAI) and Thyroidectomy

After successful management of thyroid storm, definitive treatments like thyroidectomy or RAI should be considered to prevent recurrence of thyroid storm [5]. These are considered when the thyroid storm is refractory to medical management or when ATDs are contraindicated [5]. Patients undergoing thyroidectomy should be made euthyroid by giving CBZ/MMI along with *β*-blockade [39] (and iodide for Graves' disease). The ATDs should be stopped at the time of surgery and *β*-blockers weaned following surgery [39]. High-risk patients (elderly/cardiovascular disease) undergoing RAI should be made euthyroid by giving CBZ/MMI along with *β*-blockade [3, 39]. The ATDs should be discontinued 2-3 days before RAI and restarted after 5–7 days [3], with an intention to taper down in 6 weeks to 6 months [39].

### 5.6. Prognosis

Commonest causes of death from thyroid storm were multiorgan failure, heart failure, respiratory failure, arrhythmia, DIC, gastrointestinal perforation, hypoxic brain syndrome, and sepsis [52]. Though the mortality has come down, as per the latest studies, to 3.6% from 10%, an early accurate diagnosis and multidisciplinary care are the key to the improved outcomes [1].

### 5.7. Learning Points

- Thyroid storm can be the first presentation of thyrotoxicosis
- Apathetic thyroid storm, though common in elderly, can rarely occur in young adults
- Diagnosis of thyroid storm is purely clinical, and early diagnosis improves the outcomes
- Atrial fibrillation and arterial and venous thrombosis are common in thyroid storm
- Cardiorespiratory failure is the commonest cause of mortality in thyroid storm
- PTU is the preferred ATD in thyroid storm, though it might worsen the hepatic function
- Cardio selective *β*-blockers are preferred over NCBB in thyroid storm with TCMP
- Corticosteroids (hydrocortisone or dexamethasone) should be used in thyroid storm

## Figures and Tables

**Table 1:** The Burch–Wartofsky Point Scale for diagnosis of thyroid storm [5].

Thermoregulatory dysfunction	Temperature (°C)	37.2–37.7	5 points
		37.8–38.3	10
		38.4–38.8	15
		38.9–39.3	20
		39.4–39.9	25
		≥40	30

Cardiovascular dysfunction	Tachycardia (beats/minute)	90–109	5
		110–119	10
		120–129	15
		130–139	20
		≥140	25
	Atrial fibrillation (AF)	Absent	0
		Present	10

Congestive heart failure (CHF)	Status	Absent	0
		Mild	5
		Moderate	10
		Severe	15

Gastrointestinal (GI) and hepatic dysfunction	No gastrointestinal manifestations	Absent	0
	Vomiting, diarrhoea, abdominal pain	Moderate	10
	Jaundice	Severe	20

Central nervous system (CNS) disturbance	No nervous system manifestations	Absent	0
	Agitation	Mild	10
	Delirium, psychosis, extreme lethargy	Moderate	20
	Seizure, coma	Severe	30

Precipitating event	Status	Absent	0
		Present	10 points

Total score	≥45	Thyroid storm	
	25–44	Impending storm	
	<25	Storm unlikely	

**Table 2:** The Akamizu criteria for the diagnosis of thyroid storm [5].

*Prerequisite for diagnosis*		
(i) Presence of thyrotoxicosis with high free triiodothyronine (FT3) or free thyroxine (FT4)		

*Symptoms*		
(1) CNS manifestations: restlessness, delirium, psychosis, somnolence/lethargy, coma (≥1 on the Japan Coma Scale or ≤14 on the Glasgow Coma Scale)		
(2) Fever: ≥38°C		
(3) Tachycardia: ≥130 beats/minute or heart rate ≥130 in AF		
(4) CHF: pulmonary oedema, crackles over > half of the lung field, cardiogenic shock, or class IV by the New York Heart Association or ≥ class III in the Killip classification		
(5) GI/hepatic manifestations: nausea, vomiting, diarrhoea, or a total bilirubin ≥3 mg/dL		

*Diagnosis*		

TS grade	Combinations	Requirements for diagnosis

TS1	First combination	Thyrotoxicosis and at least 1 CNS manifestation and fever, tachycardia, CHF, or GI/hepatic manifestations

TS1	Alternate combination	Thyrotoxicosis and at least 3 combinations of fever, tachycardia, CHF, or GI/hepatic manifestations

TS2	First combination	Thyrotoxicosis and a combination of 2 of the following: fever, tachycardia, CHF, or GI/hepatic manifestations

TS2	Alternate combination	Patients who met the diagnosis of TS1 except that serum FT3 or FT4 level is not available

**Table 3:** Drugs used in the management of thyroid storm [3, 6, 39].

Drug name	Treatment dose	Mechanism of action and comments
Propylthiouracil	500–1000 mg stat, then 200–250 mg every 4 hours	Blocks thyroid hormone synthesis Blocks T4 ⟶ T3 conversion (>400 mg/day)

Carbimazole	25–30 mg every 4 hours	Blocks thyroid hormone synthesis

Methimazole	15–20 mg every 4 hours	Blocks thyroid hormone synthesis

Propranolol	60–80 mg every 4 hours	Ameliorates the *β*-adrenergic symptoms

Bisoprolol	2.5–5 mg/day	Blocks T4 ⟶ T3 conversion (at high doses: propranolol >160 mg/day) Aims heart rate (HR) <130/minute Needs invasive monitoring in HF patients Asthma: use diltiazem/verapamil
Esmolol	1 mg/kg IV over 30 seconds, 150 *μ*g/kg/minute infusion	
Landiolol	1 *μ*g/kg/min as IV infusion Dose range 1–10 *μ*g/kg/min	

Digoxin	0.125–0.25 mg intravenous	Use only with normal renal function

Lugol's solution	5 drops or 0.25 mL or 250 mg every 6 hours	Blocks thyroid hormone synthesis and release. Administer 1 hour after ATD use

Lithium carbonate	300 mg every 8 hours	Blocks iodination and release

Cholestyramine	4 gm 3-4 times daily	Binds iodothyronines and removes them from the enterohepatic circulation

Hydrocortisone	300 mg IV stat, then 100 mg every 8 hours	Inhibits release and T4 ⟶ T3 conversion Prevents relative adrenal insufficiency Promotes vascular stability
Dexamethasone	2 mg IV every 6 hours	

## Data Availability

The clinical data used to support the findings of this study are included within the article.
